# Life interrupted and life regained? Coping with stroke at a young age

**DOI:** 10.3402/qhw.v9.22252

**Published:** 2014-01-23

**Authors:** Kerry Kuluski, Clare Dow, Louise Locock, Renee F. Lyons, Daniel Lasserson

**Affiliations:** 1 Bridgepoint Collaboratory for Research and Innovation, Bridgepoint Active Healthcare, Toronto, Ontario, Canada; 2 Institute of Health Policy, Management and Evaluation, University of Toronto, Toronto, Ontario, Canada; 3 Institute of Health and Wellbeing, College of Social Sciences, University of Glasgow, Glasgow, UK; 4 Health Experiences Research Group, Nuffield Department of Primary Care Health Sciences, University of Oxford, Oxford, UK; 5 National Institute for Health Research Oxford Biomedical Research Centre, University of Oxford, The Joint Research Office, The Churchill Hospital, Headington, UK; 6 Green Templeton College, University of Oxford, Oxford, UK; 7 Dalla Lana Faculty of Public Health, University of Toronto, Toronto, Ontario, Canada; 8 Nuffield Department of Primary Care Health Sciences, University of Oxford, Oxford, UK

**Keywords:** Stroke, patient experience, young, qualitative, coping

## Abstract

Stroke is a leading cause of disability across the developed world, affecting an increasing number of younger people. In this article, we seek to understand the experience of stroke as a disabling life situation among young people and the strategies that they use to recover and cope. Directed content analysis was conducted from interviews with 17 community-dwelling stroke survivors aged 55 years and younger across the United Kingdom. The sample was drawn from a larger maximum variation sample of stroke survivors. Using the sociological concepts of *biographical disruption* and *biographical repair* as a guide, excerpts from the interviews pertaining to aspects of the patients’ life that were interrupted, in addition to how they coped with the changes, were selected and analysed. All individuals described an “altered sense of self,” a theme that included loss of identity, family disruption, and/or loss of valued activities. Individuals sought to adapt their sense of self by seeking external support, by restoring normality, and/or through positive reflection. Despite the adapted self that emerged, most individuals continued to experience impairments. While young stroke survivors adapt to their illness over time, they continue to experience impairments and disruptions in their personal and work lives. A holistic model of rehabilitation that helps individuals regain the capacity for everyday activities related to work, family life, and leisure can begin to address the emotional ramifications of diseases such as stroke, restore wellness, and work towards minimizing the burden felt by family caregivers and children.

If I'd have just had my recovery to concentrate on, it would have been a lot easier than the issue of the divorce and moving house and trying to settle the children down. It's been a number of things that have all sort of come at the same time but, I mean, hey, that's life. It's like buses. You wait, don't you, and then suddenly 4 arrive all at once. (44 years, female, 3 years post stroke)

## Background

Stroke is a leading cause of disability across the developed world, affecting an increasing number of younger people (George, Tong, Kuklina, & Labarthe, 2011; Kersten, Low, Ashburn, George, & McLellan, 2002; Rothwell et al., 2005). Estimates in the United Kingdom (Kersten et al., 2002) and the United States (U.S. Centers for Disease Control and Prevention, 2012) indicate that approximately one in four individuals with stroke are under the age of 65. As noted in the opening quote, stroke at a young age can leave a particularly disabling impact given the life stage at which these individuals are.

While the literature on stroke is vast, there are few studies that capture the post-stroke experience of young people (Lawrence, 2010), and the long-term medical impact of stroke at a young age has only recently been reported (Rutten-Jacobs et al., 2013; Schaapsmeerders et al., 2013). A 2013 paper in *Neurology* noted that research on young people and stroke is lacking and that insight into factors that can reduce the burden of stroke for this population is needed (Singhal et al., 2013). The residual impacts of stroke can leave people reliant on health services for many years, a situation that is compounded by temporary or permanent loss of employment. It is estimated that only 42–53% of young stroke survivors return to work, and that of these, 23% require modifications in the workplace (Singhal et al., 2013).

In this article, we seek to understand the experience of stroke as a disabling life situation among young people and the strategies that they use to recover and cope. This description of experience is grounded in sociologist Michael Bury's conceptualization of the consequences of illness (biographical disruption) in addition to his conceptualization of how people cope with changes brought on by illness (biographical repair). The literature on young people and stroke in addition to research on biographical disruption, biographical repair, and coping in chronic illness are reviewed in brief here.

Compared to older persons, young people (under the age of 65) who have had a stroke are more likely to survive their initial illness and live many years with functional or cognitive deficits that impact their daily living (Varona, Bermejo, Guerra, & Molina, 2004). Adults who are young to middle-aged and in the midst of working and childrearing may experience a particularly profound diversion from their anticipated life trajectory. Post-stroke issues among this population include family life disruption (Daniel, Wolfe, Busch, & McKevitt, 2009; Teasell, McRae, & Finestone, 2000), marital breakup (Teasell et al., 2000), an altered sex life (Daniel et al., 2009; Morris, 2011), employment disruption (Morris, 2011) altered finances (Daniel et al., 2009; Kersten et al., 2002), and disruption in leisure activities (Daniel et al., 2009). Despite the array of issues that are experienced by young people following stroke, rehabilitation tends to solely focus on the physical aspects of recovery (Bendz, 2003; Roding, Lindstrom, Malm, & Ohman, 2003). This dichotomy between a holistic approach versus a more medical model is highlighted in Connolly and Myers’ (2003) notion of wellness, which they describe as not only physical but also psychological and spiritual. They go on to say that business and industry focus on the physical aspects of wellness alone.

A literature review of qualitative evidence of the experiences of young people and stroke conducted by Lawrence (2010) unearthed four studies. Three broad themes emerged from Lawrence's synthesis: disorientation, a disrupted sense of self, and altered roles and relationships. Disorientation was felt when individuals struggled to come to terms with physical, cognitive, and affective changes following their stroke. A disrupted sense of self (the second theme) was noted among individuals who felt little semblance to their “pre-stroke self.” The stroke itself brought on critical role changes leading to questions around identity and an urge to restore a sense of self. Finally, altered roles and relationships (the third and final theme) emerged when individuals felt that they were a burden to others. What Lawrence does not include in her review is how young people cope with stroke and the strategies they use to restore normality, a gap that our article seeks to fill. Understanding the long-term impact of stroke and the strategies that individuals use to restore their lives can inform the design of person-centred approaches to care and rehabilitation and ultimately restore a sense of wellness.

Lawrence's findings lend support, however, to existing research, particularly Bury's work on biographical disruption. In his 1982 seminal paper, “Chronic Illness as Biographical Disruption,” Bury refers to the change imposed by residual impairments brought on by chronic illness, and how this occurrence “disrupts” an individual's biography. Bury (1982) states that “chronic illness is precisely the kind of experience where structures of everyday life and the forms of knowledge which underpin them are disrupted” (p. 169). His work on people with rheumatoid arthritis revealed that diagnosis of this ailment at a young age left individuals feeling shocked and confused. Arthritis was perceived as a disease that represented the “wear and tear” of an aging body, not that of a young person. Thus, the diagnosis of arthritis at a young age marked a significant divergence from the life trajectory on which individuals perceived themselves to be prior to their diagnosis. The shock of symptoms or diagnoses can be hard at any age; however, research by Pound, Gompertz, and Ebrahim (1998)and Faircloth, Boylstein, Rittman, Young, and Gubrium (2004) suggests that older age can sometimes serve as a buffer to a chronic illness diagnosis, as it may be an “expected” part of an aging trajectory.

Similar to Bury and Pound, Charmaz (1983) examined loss of self as a result of chronic illness. Following chronic illness, she found that people tend to lead a restricted life, be socially isolated, feel discredited, and feel that they are a burden to others. However, the experience that individuals have with chronic illness is not always negative. A book by Lyons, Sullivan, Ritvo, and Coyne (1995) on chronic illness and relationships demonstrates that the experience of loss as a product of chronic illness can both compromise the stability of relationships and enhance them. The positive and negative manifestations of chronic illness are also highlighted in Frank's illness narratives (Frank, 1995), which he classifies into three types. The restitution narrative is characterized by a focus on recovery, the chaos narrative is characterized by a loss of hope, and the quest narrative is characterized as illness being an opportunity for growth. Similarly, Williams (1984) uses the term “narrative reconstruction” to describe how individuals with chronic illness establish a sense of order and meaning in their lives over time. This is similar to Bury's work and the notion of biographical repair, which occurs when individuals seek to cope with the changes in their life by reconstructing their identity and restoring a sense of normality.

There remains a limited understanding of how younger people are impacted by stroke and, particularly, how they may move on to “repair” the changes. In line with Pound's and Faircloth's assertions regarding the impact of age on the chronic illness experience, we would expect that stroke at a younger age, particularly among individuals in previous good health, would leave a particularly disabling effect.

## Method

Directed content analysis was conducted from interviews with 17 community-dwelling stroke survivors 55 years of age and younger across the United Kingdom. The interviews were drawn from a larger database of stroke survivor interviews. This larger database included community-dwelling stroke survivors who were selected for interviews using a maximum variation sampling technique (*n*=57). The set of 57 narratives were collected via individual interviews between 2006 and 2011 throughout the United Kingdom. Prior to data collection, two ethics committees (coinciding with the two data collection periods) approved this study. In 2006, it was approved by the Eastern Multi-Centre Research Ethics Committee (03/5/016), and it was approved again in 2010 from the Berkshire Research Ethics Committee (091H05051).

To recruit participants, information leaflets were distributed at stroke support groups, relevant websites, and general practitioner offices, and through personal contacts and word of mouth. Information packs were provided to stroke survivors who expressed interest. These information packs included a letter of invitation, information sheet, reply slip, and stamped addressed envelope. Those wanting to know more about the study contacted the research team. During this contact, interviews were scheduled.

The interviews were conducted by three of the co-authors (KK, CD, and LL), all trained in qualitative research. KK was trained by LL, who is the director of the Health Experiences Research Group (HERG), University of Oxford. The HERG is composed of qualitative researchers who have been conducting research consistent with the methods used in this article for over 10 years. CD is also an experienced qualitative researcher and worked with the HERG during the time of data collection. RL and DL have a long track record in stroke research from a social science and biomedical standpoint, respectively.

To begin the interview, the researchers invited the person to describe when they first noticed something was wrong. This was followed by semistructured prompting around anticipated themes. These themes were informed by the literature and the input of an expert advisory panel of laypeople, clinicians, and researchers with an interest in stroke. Interviews were transcribed verbatim and checked for accuracy before analysis. Transcripts were coded for anticipated and emergent themes using a process of constant comparison and aided by NVivo 9 data analysis software.

During analysis of the full data set of interviews, it appeared that there were specific concerns expressed by younger stroke survivors, particularly around loss of employment and relationship strain, that warranted further attention. Thus, additional analyses entailing a more in-depth round of coding of this subset of interviews were conducted by KK of the population aged 55 and under. Age 55 and under was used as the cut-off given the greater likelihood of workforce and childrearing activities within this age category. This is also the cut-off employed in the qualitative literature review on young people and stroke detailed earlier (Lawrence, 2010). Directed content analysis was used, which entails starting with a theory or relevant research findings to guide the selection of codes, which is followed by an in-depth round of coding where additional themes are captured from the selected text (Hsieh & Shannon, 2005). Directed content analysis is different from summative content analysis, the latter of which consists of counting keywords or themes. The data were thematically coded by identifying all extracts that captured the impact on stroke (including its impact on work, family and daily living, sense of identity, and loss of self), and the ways in which individuals coped with their stroke. The text was put into a coding report by KK. The text was read several times and organized into key themes. Axial coding following whereby similar themes were grouped together. Using the standard protocol of the research team, a “research buddy” (LL) independently analysed the coded data, and emerging themes were compared. Therefore, trustworthiness of the data was achieved through prolonged engagement with the data, and through discussion and comparison of codes. Confirmability of findings was enhanced by having two of the co-authors (CD and DL) read through the coding report and verify the themes. In the “Results” section, we present the findings from the analysis of the subset of interviews (*n*=17).

## Results

As indicated in Table I, six men and 11 women were interviewed; most were married with children, and the age range was 23–55 years at the time of the interview. The average age of the participants was 46.5 years. The participants were anywhere from 1 to 12 years post stroke. All of the participants were experiencing ongoing symptoms, including one or more of the following: fatigue, speech and memory impairments, mobility restrictions, partial blindness, paralysis to varying degrees, pain, muscle spasticity, and epilepsy.

**Table I:** Participant characteristics.

Interview number	Sex	Marital status and dependents	Age at time of interview	Age when stroke occurred	Ongoing symptoms from stroke
1	Female	Married, 2 children	29	28	Occasional challenges with speech
2	Female	Divorced, no children	55	43	Epilepsy, considerable loss of long-term memory, and muscle spasms
3	Male	Married, 2 children	54	53	Weakness in hand function; some vision loss
4	Male	Married, 2 children	52	47	Epilepsy, some vision loss, left-side paralysis, and mobility restrictions
5	Male	Partner, no children	50	49	Challenges with speech and mobility; partial paralysis
6	Female	Married, 2 children	31	30	Loss of visual memory
7	Female	Divorced, 2 children	44	41	Central post-stroke pain; numbness in leg and arm
8	Female	Married, 1 child	41	34	Hemionaopia and epilepsy
9	Female	Divorced, no children	54	48	Aphasia and epilepsy
10	Male	Married, 4 children	47	45	Arm paralysis, pain, and muscle spasms
11	Female	Married, 2 children	54	32 and 52*	Arm and leg weakness and memory problems
12	Female	Single, no children	34	29	Left-side paralysis, aphasia, and epilepsy
13	Male	Married, 1 child	44	38	Aphasia and right-side paralysis
14	Female	Single, no children	23	21	Minor speech and memory impairments
15	Female	Single, no children	28	26	Fatigue
16	Female	Married, no children	50	46	Loss of mobility
17	Male	Married, 2 children	47	45	Hemianopia, mobility restrictions, uncontrolled muscle spasms, and left-side paralysis

* Recurrence.

Two broad themes were gleaned from the transcripts: an altered sense of self and an adapted sense of self. The first theme (altered sense of self) had three subthemes: loss of identity, family disruption and role change, and loss of valued activities. Illustrative quotes are provided under each theme. Transcription conventions include [text within square brackets] to provide clarification as well as […] to illustrate omitted text that was deemed redundant.

### Altered sense of self

Following the stroke, all individuals felt that some aspect of their self had been disrupted. Common challenges included a compromised ability to perform activities of daily living (e.g., eating, moving, and bathing) in addition to problems with speaking and remembering. These challenges, for some, improved over time; however, the lasting impact tended to manifest as a discredited notion of self. Individuals perceived themselves differently due to changes in their characteristics, their ability to fulfil certain roles (e.g., in the family or workplace), and their ability to engage in activities that were important to them, including being part of meaningful social relationships. All of these findings are explored in detail here. We start with the first subtheme, “loss of identity.”

#### Loss of identity

Many individuals were shocked that they had a stroke at such a young age, particularly if they perceived themselves to be in relatively good health beforehand. A 41-year-old woman who had an ischaemic stroke at the age of 34 (which caused paralysis, hemianopia, and the onset of epilepsy) described the shock of having a stroke.

[A] lot of the sort of, “why me?” started to creep in, particularly because I was so fit, hadn't drunk, hadn't smoked, lived at the gym virtually, blood pressure was always bang on normal. (Participant #8)

In a couple of cases, the shock of dealing with the stroke was worsened by a delay in receiving a diagnosis, as medical staff felt that the person was too young to be experiencing such an event. One woman, who experienced visual disturbance, was thought to be taking drugs or alcohol.

Following the shock of diagnosis, many individuals described feeling a sudden loss of their former selves. One respondent noted, “[It's] as if somebody came in the middle of the night on the 16th of December and stole part of me and I have never got it back.” (Participant #7)

Similarly, a woman who had two strokes over a 3-month period at age 45 described her stroke as bereavement, and pinpoints the precise date.

…it really is like a bereavement, a stroke. I mean, it really was like that woman had died, the one that wore high heeled shoes and walked around and ran a business and, had a fantastic memory. I had one of the most amazing memories that I took totally for granted. There's so much that one can take for granted, you know, I take walking for granted. I always think, “If only somebody had told me on New Year's Eve 1995 that that's the last hour I would have ever walked like a normal person because I would have walked all day…” (Participant #2)

Some people felt as if they had reverted back to being a child, as if they were “a baby with an adult mind.” Even simple daily tasks, like taking the city bus, became an unnerving process. A woman who had a stroke at age 48 that caused aphasia and epilepsy shared her difficulty:

…and then eventually I thought, right now I've got to get up and I've got to press something and say “I'd like to get out now” but when I came out towards the door, I was terrified. I thought my legs were coming out too quick. It was fine actually but oh, it took me weeks and weeks to do it properly. I'm much better now and of course I find, I still find it very difficult when I have difficulties about going in the right directions. (Participant #9)

Losing the ability to fulfil activities of daily living and take on multiple tasks hindered motivation, patience, and confidence. This was particularly difficult for people who were used to juggling a range of activities, including family life, a regular exercise regimen, and work. A woman with two children who had a stroke at the age of 30 shared the following:

I can't multi-task now and that's hard when I'm looking after the kids. That's hard for work as well. I used to be able to fly round doing about seven or eight things at once. Now, I try to get one thing done and then something happens and I need to prioritize something else… (Participant #6)

In summary, the shock of diagnosis, the initial symptoms, and the impact on day-to-day functioning (which was once second nature) were met with much frustration.

#### Family disruption and role change

Many respondents noted changes in family roles following the stroke. A woman who had a stroke 18 months after her child was born had “very little recollection of her [daughter] as a tiny baby.” For other respondents with young children, not being able to read to them, embrace them, or pick them up from school became difficult realities. In some cases, individuals were encouraged to hold off on having children following their stroke. A young woman who was interviewed with her husband, a stroke survivor, explained that they decided not to have a second child. In another case, a young woman was told by healthcare providers to delay additional childbearing after experiencing a stroke 4 months after giving birth. For these individuals, a clear divergence was made from their life plan.

In addition to childrearing interruptions, the stroke had an impact on family relationships. A man with a wife and two young children who had a stroke 2 years before the interview at age 45 talked about the impact on his family.

And the initial shock of coming home actually did remain for quite a long time because the psychological effect on the kids of seeing my condition and that I couldn't be the dad that perhaps I once was in terms of physically playing with them and doing things. I couldn't even pick them up because I could only use one hand, one arm that was quite hard on them. And the kids had quite a lot of counselling. And my wife and I had quite a bit of counselling. (Participant #17)

In some cases, the spouse or partner had to take time from work (temporarily or permanently) to fulfil caring duties (of the person with stroke and/or of children). Respondents were embittered that “they [family] have had to learn to live with my deficits as well as myself and they've had to learn to adapt their lives.” (Participant #7)

People in this age group will often have dual-facing care responsibilities, with not only their own children but also aging parents looking to them for support. An unanticipated reversal of care responsibilities between generations was noted by one woman who said that she should be caring for her aging parents, and not the other way around. Younger stroke survivors might need support from their parents not only for themselves but also for their children, with grandparents suddenly placed in a parenting role again.

Some of the participants indicated that their disability compromised the romantic side of their relationships. Consider this married father of four children who had a stroke at the age of 45:

It would be great to be able to go on a holiday, just hold hands and walk along a beach or just go for a picnic out in the country and sit by the river [...] But if we do anything like that, I've got to rely on my wife to push me in the chair, which I don't think sounds very romantic then, having to be taken rather than me taking my wife… (Participant #10)

Similarly, a younger woman in her late 20s who had a transient ischemic attack (TIA) followed by a full-blown stroke was concerned about her future romantic relationships:

I think relationship-wise when you first meet somebody and say, “Oh yeah, I had a stroke when I was 26” that's kind of hard for somebody to get their head round and they kind of run away from it a bit… (Participant #15)

In summary, disruptions to childrearing plans, changes to spousal and family relationships, notable impacts on others in the family (e.g., spouses taking time from work, and children seeking counselling), and concerns about the quality of future relationships were noted.

#### Loss of valued activities (work, recreation, and socialization)

Loss of valued activities included disruption to tangible activities (e.g., employment, recreational activities, and driving) as well as relational activities (e.g., socializing with friends). These activities were commonly described as integral aspects of self. These activities were described as not only enjoyable but also, in the case of employment, financially necessary. A divorced mother and former fitness instructor with two children who had a stroke resulting from a previously undetected heart problem explained how the stroke was a double blow to her identity:

It's the fact that my sport was my life, it was my pleasure, it was my job, financially, everything.... every spare moment of the day was devoted to aerobics or pilates or yoga or whatever I chose to do, whilst the children were at work and, at school, and I, I lost it just like that with, with literally the turn of a page or a, a breath, that's all it was. It was a breath while a clock moved upwards. (Participant #7)

This reference to a pinpoint in time—“a breath while the clock moved upwards”—represents the sudden breach with an active self.

Loss of employment, whether temporary or permanent, was noted frequently. For example, some participants had to close a self-owned business while others were made redundant post stroke. Some individuals had already returned to work, albeit in a modified capacity. In most cases, work disruption had clear financial consequences due to loss of revenue, which, for some, was compounded by additional expenses required for home adaptations to accommodate their disability. A married mother with two children who suffered from two subarachnoid haemorrhages shared the following:

…it was a big impact because we'd bought our first house prior to the subarachnoid and we needed both wages with the mortgage repayments being so high and then falling pregnant 6 months after the sub-arachnoid obviously meant that it was going to be longer before I could actually go back to work again so, yes, it had a, a very big financial impact. (Participant #11)

In addition to the costs and impact related to loss of employment, loss of ability to partake in recreational activities was noted. For some, recreation (i.e., fitness) was a defining part of their identity that stroke threatened. A man who had a stroke at the age of 53 during a heart operation shared the following:

…obviously you're thinking ahead, months and months ahead, “Is this how it's going to be?” You know, “As I say, I've run marathons, half marathons, climbed hills and played golf and cycled” and I was thinking ahead, you know, “Is this how it's going to be?” (Participant #3)

A 45-year-old man who had his license revoked described a similar sense of loss:

…they wrote to me to say that in the March, like in 2 weeks’ time, they were revoking my license. I mean, I wasn't up to driving anyway and, but that was quite annoying that happened because I'd been driving since I was 16 and, you know, it's, oh, it was a big part of my life when you have to use public transport with a toddler. (Participant #11)

In addition to the loss of the more tangible activities (i.e., work, recreation, and driving), disruptions to social-relational activities were noted. For some people, the ability to socialize was compromised due to functional limitations like fatigue. A single woman with no children, who had a stroke at the age of 29 leaving aphasia, paralysis on her right side, and epilepsy, noted the following:

Sometimes sad with, you know why, you know, out of work, you know, socialising, you know, and I'm tired, exhausting because, you know getting up, shower, takes time…. (Participant #12)

Some participants distanced themselves from social relationships and outings because it was a reminder of their former self. Others were concerned that they would be perceived to be “not as fun anymore” or self-centred due to memory issues and a lack of ability to engage in meaningful, ongoing conversation.

In this section, we demonstrate that stroke left people unable to fully engage in their hobbies, work, and social activities, which were regular aspects of their lives previously.

### Adapted sense of self

For many people, the stroke caused seemingly catastrophic alternations to their lives; however, this was frequently countered by efforts to adapt and move on with life in its new form. This adapted sense of self is described by the following three subthemes: seeking external support, restoring normality, and positive reflection.

#### Seeking external support

Despite the changes in social relationships and family roles noted in the themes above, some respondents, including those who initially felt isolated from family and peers, talked about the important roles of family, friends, peer support groups, and employers in the recovery process. A woman who had an ischaemic stroke at the age of 28 noted the following:

I have had the most tremendous support from my family and friends and I think that has just made the biggest difference to me. Everybody around me has just been so good I think. That's really helped me with my overall outlook on things that I've just kind of just got to get on with it. (Participant #1)

Among participants who had to take time from work, some were able to return in a modified capacity following a period of recovery. A woman who had a stroke at the age of 48 described the process of work reintegration, and how the allowances made by her employers enabled her to participate in the workplace in a modified way.

I mean, they were very good to me and I could, when I went back part-time, they let me pick and choose what hours I could manage and I just, because I, I did get very tired, particularly in the afternoons, so often I'd go in the mornings and perhaps work till perhaps 1 o'clock and come home because that's all I could manage. (Participant #8)

Despite the initial disruption and stress associated with work and family, these very spheres became important sources of support.

#### Restoring normality

A few individuals articulated their post-stroke experience as akin to being a new person. Removing aspects of their old self became a form of coping. A woman who experienced two haemorrhagic strokes 3 months apart shared the following:

I had long hair […] I had all my hair cut off. Right off, while staying with a friend. I mean, really, really short. The hairdresser said, “Are you sure about this?”, but it was because in a way I thought, “Well, this hair is the hair of her, it's the hair of the woman, that could, that walked and was well and wasn't, you know, and, wasn't disabled and I don't want her hair anymore because I'm not her now. I'm somebody new.” (Participant #2)

Many individuals, including those who initially sought to create a “new self,” tried to restore their sense of self by engaging in activities that were important to them before the stroke such as visual arts, music, or public speaking. A woman who had two haemorrhagic strokes 3 months apart used her passion for piano to facilitate active physical rehabilitation.

I played the piano, from the age of 7, it was almost innate to me […] so I had to find a way into my brain. So what I did was to imagine a picture of a piano keyboard in my head. I always closed my eyes and I could see this keyboard and, with my mind, I would make my mind press a note down that I could see, and the first time I did it, I got a tiny, tiny movement in my forefinger, just miniscule. And I thought, “Goodness me”, and I pressed the note down again and I got a bit more movement and a bit more... So I practiced and practiced on what I called neural notes basically… (Participant #2)

During a process of recovery, many people sought to re-establish roles to ease the burden on their loved ones by helping out around the house, taking the kids to school, and preparing meals. As articulated by one man, he did this so he would “not just be a useless blob around this house that can't do anything” (Participant #17).

In addition to re-establishing roles in the household, equally important was re-establishing a role in the workplace. One woman talked about what it meant to her to get her job back, even though it was a different job than she had before her stroke.

I sat down with a blank piece of paper and thought, “Well, what do I want to do?” I knew I didn't want to stay at home all the time. I quite enjoy meeting people and seeing people and being part of a team, if not leading people, so I decided to go and see an old friend to ask her to be my second reference if I applied for something and she offered me a job so I've been there ever since. (Participant #11)

In summary, restoring normality was an important part of the recovery process. Re-establishing roles in the household and workplace and reverting back to old hobbies were common among the study participants.

#### Positive reflection

As respondents settled into their lives post stroke, it was typical to go back and forth between grieving for their former capabilities and positively reflecting on their progress. A woman who had an ischaemic stroke at the age of 30 talked about feeling resentful as well as positive since her stroke.

...but you're going to feel all different ways. You're going to feel resentful, why me? You're going to feel down in the dumps, you're going to feel angry, you're going to feel sad, you're going to feel frustrated, you're going to feel absolutely everything under the sun and it's just, it's just the process of accepting it and getting better, getting stronger. (Participant #6)

As a first step, a woman who had a stroke at the age of 53 during a major heart operation talked about the importance of accepting dependence as part of adapting to it.

…you're going to the toilet, you know, and they're [medical staff] actually cleaning you up, you know, and you're thinking to yourself, “God, this is terrible.” But they're actually saying at the time, “Don't worry, this is, this is our job, we do this every day, we'll do it for years to come, so don't worry.” And once you get that in your mind, once you get that implanted in your mind and accept it, to me, that is a big, big hurdle to get across. (Participant #3)

Some participants expressed gratitude for being given a “second chance” or told themselves that it “could have been worse.” To facilitate recovery, a couple of participants drew on stories of others who had overcome hardships, using downward social comparison with people they perceived to be worse off than they were to motivate themselves. One respondent noted:

And he [Douglas Bader] actually was quite a successful Spitfire pilot. Shooting down lots of German planes when he only had two legs. And there's been times in my life when I've need to get up the steps or try and use a lawnmower or anything and I just think in my mind…Douglas Bader, Douglas Bader. “If he can get in a plane and fly with two wooden legs, then I can do anything.” (Participant #17)

For younger stroke survivors, wanting to continue to meet parental responsibilities and goals, such as “wanting to see the children grow up and settle down and have families of their own,” could provide an added spur. Similarly, another respondent reflected “that looking after my two kids and being a mother to them, being a mum, doing what mums do, that's an achievement to me.” (Participant #6)

In summary, people reflected positively on their progress and expressed gratitude for having the opportunity to continue on their life trajectory, despite ongoing hardships.

## Discussion

The purpose of this study was to understand the impact of stroke on young survivors in addition to how they cope with the changes. While there is a breadth of literature on the experience of having a chronic illness, including the impact on personal and social identity (Bury, 1982; Charmaz, 1983, 1987, 1991, 1994, 1995; Lawrence, 2010) and the process of restoring a sense of being (Locock, Ziebland, & Dumelow, 2009; Williams, 1984), little attention has been paid to the experience of stroke as an event that interrupts a young person's biography and the strategies used to cope with these changes. Understanding these strategies can inform the way in which care is designed for this population.

Individuals in our study were typically shocked by their diagnosis and had serious questions about their identities, typically in the initial phases following stroke. Some felt like a different person altogether, while others noted certain aspects of self that had changed. Frustrations at losing certain abilities such as multitasking, which is critical to juggling work, childrearing, and other social activities, were common. This “discredited notion of self” (Charmaz, 1983) played out heavily as individuals contended with the changes in their lives and identities.

The impact that the stroke had on family, marriages, and social relationships was noted. Although the intent of the article was not to conduct an analysis by sex, we did see some subtle differences in this theme. Family planning and the ability to attract a future partner were themes identified by women only. The women in our sample were disproportionately represented in the younger age cohorts of our sample, which may be why the topic did not surface among men. The men tended to emphasize changes to their household roles and concern over the extra burden that was put on the shoulders of their partners and the resentment that might ensue. No obvious sex differences were noted in workforce disruption. Many men and women had to quit their job or were made redundant following their stroke, which bore huge financial consequences and shook personal identities. The impact extended to spouses and partners, who had to take on additional responsibilities which sometimes entailed leaving the workforce as well.

In terms of coping or seeking repair, much resilience was demonstrated across the whole sample in terms of positive attitudinal shifts, restoration of some aspect of self, and making small continual gains. Individuals in our sample were interviewed anywhere from 1 to 12 years after their stroke, with all study participants experiencing continued impairments, albeit to different degrees. There did not appear to be a consistent relationship between the time that elapsed since stroke and the extent of recovery. For example, while one person felt almost fully restored 2 years after their stroke, another person had ongoing impairments 7 years later. To that end, stroke manifests in many different ways, with residual impacts extending over many years or a lifetime.

Stroke rehabilitation tends to be concentrated in the first 6 months following a stroke, but our data, much like other research, highlight that stroke recovery is a much longer process, characterized by periods of stability and periods of uncertainty. For example, a survey conducted by the Stroke Association in the United Kingdom found that services are lacking after individuals leave hospital (Stroke Association, 2012). Half of the individuals surveyed (53%) had been assessed only once after being discharged from hospital, and coordination of care had fallen largely on the shoulders of family.

Young stroke survivors have stated that care services tend not to be congruent with their needs post stroke (Bendz, 2003; Roding et al., 2003). There have been clear advances in hospitalized stroke care to address acute symptoms; however, there remains a lack of focus on post-stroke interventions that address psycho-social symptoms, including the manifestation of depression and stress that may hinder social and workforce participation (Lyons, Rudd, & Alvaro, 2007). A literature review conducted by Murray, Young, Forster, and Ashworth (2003) revealed that the most common long-term problem identified by stroke patients and their carers was lack of emotional support.

Our findings have important implications for the role of rehabilitation in post-stroke recovery. We draw on three key examples from our findings as important considerations for post-stroke rehabilitation services: workplace reintegration, mental imagery, and psychosocial support for the person with stroke and their family.

### Workplace re-integration

Many individuals identified themselves through their work and leisure activities, and they were at a loss when these activities were no longer possible or had to be modified in a substantial way. Getting back to work was a huge concern for many of the participants, and much gratitude was expressed to employers who allowed them, in some cases, to return to work, typically in a modified capacity. Some individuals with more lasting impairments (i.e., issues with ongoing pain, mobility, or cognitive changes) were unable to return to work and were suffering not only physically but also financially. The Canadian Best Practice Guidelines Recommendations for Stroke Care (Lindsay et al., 2010) outline the importance of community re-integration following stroke and recommend support for vocational re-integration in addition to ongoing assessment of risk factors, management of comorbid conditions, and assessment of rehabilitation needs. The extent to which this has been rolled into regular practice is unclear. Likewise, a recent article published in the *British Medical Journal* noted that workplace re-integration should start earlier in the rehabilitation process and that care providers should play a role in encouraging early communication between patients, families, and employers (Frank, 2013).

### Mental imagery

It is interesting to reflect that in our data, we demonstrate self-generated rehabilitation interventions in the use of mental imagery to improve motor function. This manifestation of internally driven repair has been the subject of research trials (Ietswaart et al., 2011), and other informal rehabilitation techniques have been observed in resource-poor health settings (Jullamate, de Azeredo, Paul, & Subgranon, 2006). The wider health practitioner community may have more to learn from the qualitative paradigm about successful forms of rehabilitation in young patients with stroke. Of interest, the person who used mental imagery in our study also personalized her recovery by envisioning a piano in her mind, drawing on her skill as a pianist. Such personalization of care may represent a way to minimize the notion of self-loss that characterizes the post-stroke experience.

### Ongoing emotional support for the person and their family

Many individuals experienced an attitudinal shift during their post-stroke trajectory, characterized by positive reflection and acceptance; however, there was a tendency for individuals to move back and forth from grieving to being as once was, to making modifications and accepting the changes. These stages relate to Frank's illness narratives (Frank, 1993, 1995): restitution (focus on recovery), chaos (loss of hope), and quest (an opportunity for growth). The chaos narrative appeared to be more prevalent among individuals with more lasting impairments, where it seemed that they experienced periods of frustration mixed with moments of hope. This suggests that the emotional impact of stroke is less predictable and requires ongoing attention throughout the years following stroke.

Concern over the impact on children and spouses was also of heightened concern, and this aligned with the notion of “becoming a burden” (Charmaz, 1983). It became clear to individuals that stroke impacted not only them but also others within their family and social networks. Finding ways to minimize that strain was of utmost importance. Persons with stroke engaged in strategies to minimize household burden, including taking children to school or helping prepare a meal. Some of the stroke participants were acutely aware of the stresses and strain put on their spouse or partner as they took on additional responsibilities; thus, any contribution to the household appeared to ease their feelings of angst.

Leading restricted lives (Charmaz, 1983) occurred for some people who had a hard time maintaining their previous social life. For instance, some people distanced themselves from friends because it served as a reminder of their former selves. Others worried that they were being a burden, and shared concern over the changing dynamics of their relationships with their friends, parents, spouse or partner, or children. These findings point to the importance of continual psychosocial support for persons and families following stroke, including strategies to help persons re-engage in their social networks and maintain social capital; these are critical for ongoing health and wellness.

## Conclusion

The uniqueness of stroke from other chronic illnesses is its sudden and often unexpected onset followed by lasting impairments across physical, cognitive, and affective domains. When individuals are young to middle-aged, stroke-related impairments severely disrupt an important part of their life trajectory, which is typically characterized by workforce participation, childrearing, and other meaningful forms of social participation. In our study, the stroke also disrupted the lives of the individual's familial network, as exemplified by spouses who released themselves from employment activities and children who had to adapt to the role changes of their parents. Here, the cost to the family as well as the system (the person and their spouse exiting the workforce at midlife) was significant. A holistic model of rehabilitation that helps individuals regain the capacity for everyday activities related to work, family life, and leisure can begin to address the emotional ramifications of diseases such as stroke, restore wellness, and work towards minimizing the burden felt by family caregivers and children. A key goal of our study population was going back to work and remaining active in their social and family lives. An incredible amount of resilience was demonstrated in our study population, which was mixed with feelings of uncertainty and frustration. These is an opportunity to capitalize on this readiness for change through a long-term rehabilitative strategy designed to reintegrate individuals back to some aspect of their pre-stroke self sooner than is occurring at present.

## Limitations

The interviews were conducted by three of the co-authors (KK, CD, and LL), which may have produced variation in the findings. While all interviews started with the general question “Tell me about your experience,” further probing and questions may have been partly biased by the interviewer's personal insights and interests. Furthermore, given that individuals are recruited via websites, newsletters, and so on and had to contact the department where the researchers were housed to schedule an interview, this may have led to selection bias towards higher functioning stroke survivors. Therefore, our finding that all individuals moved to restore their sense of self, albeit in a variety of ways, is not necessarily transferable to other stroke survivors. Nonetheless, the findings from this research shed light on a relatively neglected experience, suggest directions for further research, and have important implications for the design of rehabilitative care.
